# Fluid-induced, shear stress-regulated extracellular matrix and matrix metalloproteinase genes expression on human annulus fibrosus cells

**DOI:** 10.1186/s13287-016-0292-5

**Published:** 2016-02-27

**Authors:** Po-Hsin Chou, Shih-Tien Wang, Meng-Hua Yen, Chien-Lin Liu, Ming-Chau Chang, Oscar Kuang-Sheng Lee

**Affiliations:** School of Medicine, National Yang-Ming University, Taipei, Taiwan; Department of Orthopedics and Traumatology, Taipei Veterans General Hospital, Taipei, Taiwan; Institute of Clinical Medicine, National Yang-Ming University and Academia Sinica Taipei, Taipei, Taiwan; Department of Medical Research, Taipei Veterans General Hospital, Taipei, Taiwan; Taipei City General Hospital, No.145, Zhengzhou Rd., Datong Dist., Taipei City, 10341 Taiwan (R.O.C.)

**Keywords:** Intervertebral disc, Annulus fibrosus, Mechanical loading, Shear stress, Bio-microfluidic device, Mechanobiology

## Abstract

**Background:**

Mechanical loading plays an important role in the regulation of extracellular matrix (ECM) homeostasis as well as pathogenesis of intervertebral disc (IVD) degeneration. The human annulus fibrosus (hAF) in the IVD is subjected to contact shear stress during body motion. However, the effects of shear stress on hAF cells remain unclear. This aim of the study was to investigate the expression of the ECM (*COLI*, *COLIII* and *aggrecan*) and matrix metalloproteinase (*MMP-1*, *MMP-3* and *ADAMTS-4*) genes in hAF cells following fluid-induced shear stress in a custom-fabricated bio-microfluidic device.

**Methods:**

hAF cells were harvested from degenerated disc tissues in routine spine surgery, staged by magnetic resonance imaging, expanded in monolayers and then seeded onto the bio-microfluidic device. The experimental groups were subjected to 1 and 10 dyne/cm^2^ shear stress for 4 h, and no shear stress was applied to the control group. We used real time polymerase chain reaction for gene expression.

**Results:**

Shear stress of 1 dyne/cm^2^ exerted an anabolic effect on *COLI* and *COLIII* genes and catabolic effects on the *aggrecan* gene, while 10 dyne/cm^2^ had an anabolic effect on the *COLI* gene and a catabolic effect on *COLIII* and *aggrecan* genes. The *COLI* gene was upregulated in a stress-dependent manner. Expression of *MMP-1* was significantly higher in the 10 dyne/cm^2^ group compared to the control group (*P* < 0.05), but was similar in the control and 1 dyne/cm^2^ groups. Expression of *MMP-3* and *ADAMTS-4* were similar in all three groups.

**Conclusion:**

Taken together, hAF cells responded to shear stress. The findings help us understand and clarify the effects of shear stress on IVD degeneration as well as the development of a new therapeutic strategy for IVD degeneration.

## Background

The intervertebral disc (IVD) is composed of two distinct components [1, 2], the central gelatinous nucleus pulposus (NP) and the surrounding outer annulus fibrosus (AF). Movement of the spine subjects the bony structure of the spine and the IVD to mechanical loading [2, 3]. Degeneration of the IVD, which has been identified as one of the major sources of back pain [4], has a complex pathogenesis [1, 5]. A number of mechanical factors such as tension [6], compression [7], shear stress [3] and vibration [8] as well as other factors such as aging, genetic and systemic factors have been implicated in the pathogenesis of IVD degeneration [5].

IVD degeneration is characterized by increased degradation of the extracellular matrix (ECM) by locally produced matrix metalloproteinases (MMPs) and aggrecanase which belongs to the ADAMTS family (a disintegrin and metalloproteinase with thrombospondin motifs) [1, 9]. Collagens and aggrecans, which are the major components of the ECM in the IVD, are synthesized by the IVD, and broken down by MMPs and aggrecanases [5] to achieve dynamic equilibrium.

In vitro experiments demonstrated that proper mechanical loading plays an important role in the function and metabolism of IVD cells [2, 6–8, 10]. Epidemiologic studies [11] also revealed that mechanical overloading resulting from bending, twisting and heavy lifting were important risk factors for back pain, IVD degeneration and herniation [5, 11].

IVD degeneration has been shown to include different types of human AF tears or defects (peripheral, circumferential or radiating) [12]. Although AF degeneration seems to occur following NP degeneration [13], biochemical changes in AF are accompanied by IVD degeneration, suggesting that AF may also play an important role in IVD degeneration [12], particularly the initiation of IVD degeneration.

During movement of the spine, the IVD and human AF (hAF) are exposed to multiple, complex, three-dimensional mechanical loading forces, including compression, tension, lateral bending, vibration, shear stress or a combination of these [5, 14]. The change in intradiscal hydrostatic pressure during spine movement also impacts tissue fluid influx and efflux within the IVD [15]. Consequently, hAF cells are constantly subjected to fluid-induced shear stress even without body motion [3]. Fluid-induced shear stress was defined as the tangential component of frictional forces generated at a surface by the flow of a viscous fluid, reported by Chiu and Chien [16]. Although there is growing awareness of the role of mechanical loading on the metabolism of human, caprine and rabbit AF cells [6, 8, 17], the impact of shear stress on hAF cells has yet to be explored.

We hypothesized that shear stress might play a vital role in the expression of ECM and metalloproteinase genes in hAF cells. We investigated the effects of fluid-induced shear stress on the expression of ECM genes (*COLI*, *COLIII* and *aggrecan*), and MMP genes (*MMP-1*, *MMP-3*, and *ADAMTS-4*) of hAF cells harvested from degenerated disc tissues in a custom-fabricated bio-microfluidic device.

## Methods

The study was approved by the ethical committee of the Taipei Veterans General Hospital prior to the study. Informed consent for using the disc tissue for the study was obtained before the index operation. Disc tissues were obtained from three female and three male donors (mean age 55 years, range 47–62 years) during disc excision for transforaminal interbody fusion (TLIF) (n = 6) (Table 1). The degenerative status of the discs was classified based on Pfirrmann’s classification using T2-weighted magnetic resonance images [18]. hAF cells were isolated from AF tissues obtained from IVD after TLIF surgery. The most inner and outer layers of AF tissues and cartilaginous endplates were carefully removed, and only the middle layer of AF tissues was used for further enzymatic digestion. hAF tissues were separated from the discs immediately after surgical excision, finely minced, and transported under sterile conditions to the laboratory for primary hAF culture. To obtain hAF cells, tissues were gently agitated in the presence of phosphate-buffered saline (PBS) containing 0.1 % collagenase for 30 min at 37 °C and then at room temperature for another 30 to 40 min. After digestion, the fragmented tissues were passed through filters, centrifuged twice, and cells were resuspended in Dulbecco’s modified Eagle medium (DMEM; Sigma-Aldrich) supplemented with 10 % fetal bovine serum (FBS; HyClone, Logan, UT, USA), 100 U penicillin, 1000 U streptomycin, 2 mm L-glutamine (Invitrogen, Carlsbad, CA, USA), and 1 ng/mL recombinant human fibroblast growth factor-2 (FGF-2; Sigma-Aldrich). The cell suspension was plated in dishes and incubated at 37 °C in a humidified atmosphere of 5 % CO_2_ and 95 % air. Culture medium was changed twice a week.

**Table 1:** Patient demographic data

	Age (years)	Gender	Diagnosis	Operation
Patient 1	62	Male	L 45 listhesis	L 45 interbody fusion
Patient 2	55	Male	L 345 listhesis	L 345 interbody fusion
Patient 3	47	Male	L 45 listhesis	L 45 interbody fusion
Patient 4	60	Female	L 345 listhesis	L 345 interbody fusion
Patient 5	52	Female	L 45 listhesis	L 45 interbody fusion
Patient 6	54	Female	L 45 listhesis	L 45 interbody fusion

*L* Lumbar, *listhesis* Spondylolisthesis

When hAF cells from degenerated disc tissue (passage 0) were 70–80 % confluent, cells were detached with 0.25 % trypsin–ethylenediaminetetraacetic acid (Gibco BRL), washed twice with PBS, centrifuged at 1000 rpm for 4 min, and cultured in T-75 flasks as described previously (passage 1). When hAF cells reached 80 % confluence, they were harvested and expanded in T-75 flasks (passage 2). Passage 2 of hAF cells which were 80 % confluent were detached with trypsin, washed twice with PBS and cryopreserved in liquid nitrogen in FBS with 10 % dimethyl sulphoxide (DMSO). All experiments were performed with cells from passages 2 and 3 in order to avoid de-differentiation during culture over long periods (p < 6) [19].

**Fig. 1:**
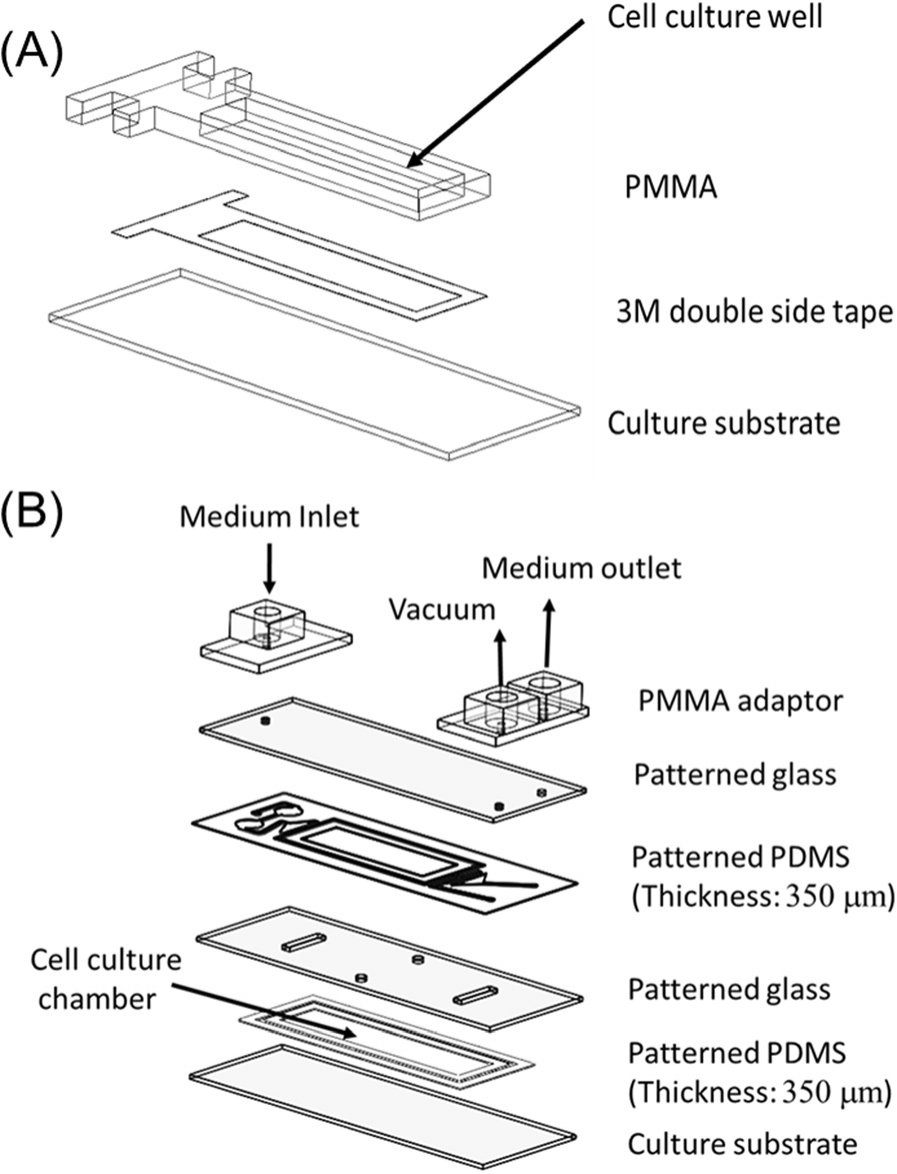
Design of the custom-fabricated microfluidic device. **a** Schematic representation of the “pre-culture well”. **b** The assembly of the microfluidic device with a cross-sectional view of the structure. *PDMS* Polydimethylsiloxane, *PMMA* Polymethyl methacrylate

We used a modified prototype of a custom-fabricated microfluidic device [20]. The five-layer device comprised a culture chamber, a vacuum region, and bubble traps. The width of the culture chamber was 10 mm, the length was 40 mm and height was 350 μm. The bubble-removal structure in the micro-channel prevented bubbles from being injected into the culture chamber. The microstructure was fabricated with a polymethyl methacrylate (PMMA) adaptor, two layers of glass, and two layers of polydimethylsiloxane (PDMS) (Fig. 1). The top layer of the device, consisting of three adaptors to produce a vacuum, and the medium inlet and outlet, was made of PMMA. The intermediate layer of the device was made of two patterned glass layers and two patterned PDMS layers. PDMS prepolymer (Sylgard 184, DowCorning) was prepared by mixing PDMS base with a curing agent at a 10:1 ratio by weight. The PDMS prepolymer mixture was then poured on the glass mold to a thickness of 350 μm and cured at 60 °C in an oven. Next, the PDMS membrane was patterned using a CO_2_ laser machine (ILS-II, Laser Tools and Ttechnics Corp.) and the glass was patterned with an ultrasonic drill machine (Lapidary & Sonic Ent.) to make the microfluidic channel, the culture chamber, and the vacuum channel. The microfluidic device was formed by bonding the patterned glass and PDMS using a plasma treatment system (PX-250, Plasma treatment System, America North) and attaching this to the PMMA adaptor using 3 M tape.

Rhodamine 6G was used as a tracing dye to ensure that the flow field on the microfluidic device was uniform and that the flow of the medium into the cell culture region was homogeneous. The flow field in our custom-fabricated chip has been shown to be uniform and steady [20]. Human mesenchymal stem cells (Lonza, PT-2501) were used to test biocompatibility in further experiments.

Fibronectin (Fn) (bovine; Sigma-Aldrich) was diluted in PBS to a working concentration of 100 μg/mL. Diluted Fn (500 μg) was loaded smoothly onto the custom-fabricated bio-microfluidic device and incubated at 37 °C for at least 1 h. The Fn solution was then removed, and the device washed twice with PBS.

**Fig. 2:**
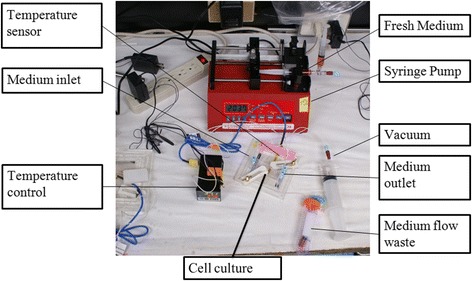
Bio-microfluidic device used for the fluid-induced shear stress experiment

hAF cells (8–10 × 10^4^) were seeded onto the fabricated laser-cut dishes and incubated overnight at 37 °C. The microfluidic device was sterilized by injecting it with 75 % ethanol via the plastic pipes and exposing it overnight to ultraviolet radiation. The laser-cut dishes were assembled with the custom-fabricated bio-microfluidic device under sterile conditions. The pumping machine was set up with a 50 mL syringe filled with medium as fluid-induced shear stress from the inlet. The device was placed on a thermal control plate at 37 °C. During the experiment, medium supplied via the 50 mL syringe was injected by the syringe pump into the chamber of the microfluidic device through the pipeline (Fig. 2). There was a continuous flow of medium from the inlet into the microfluidic device, and spent medium flowed out from the outlet to the waste tube. Based on the cell-in-channel model adapted by Farokhzad et al. [19] and Gaver and Kute [21], the maximum shear stress in our chip was calculated to be 1 dyne/cm^2^ when the flow rate was set to 10.14 mL/h.

**Fig. 3:**
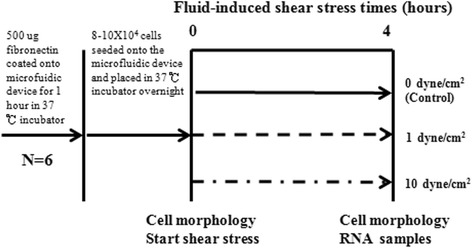
Experimental flowchart. Degenerated hAF cells (n = 6) were seeded onto the bio-microfluidic device and incubated overnight. RNA was extracted from each plate and gene expression evaluated by real-time polymerase chain reaction

Continuous fluid-induced shear stress of 1 and 10 dyne/cm^2^ was applied through the microfluidic device for 4 h, respectively. The control group consisted of hAF cells in the device not subjected to shear stress (0 dyne/cm^2^) (Fig. 3).

The expression of different genes following fluid-induced shear stress was evaluated by quantitative real-time polymerase chain reaction (PCR). Total RNA was extracted from 8–10 × 10^4^ cells using the RNeasy Mini Kit (Qiagen, Stanford, Valencia, CA, USA). RNA samples were used for reverse transcription and subsequent PCR amplification. Quantitative real-time PCR was performed using the LightCycler 480 Real-Time System (Roche Applied Sciences, Mannheim, Germany). Intron spanning primers specific for each targeted gene were designed using the Universal ProbeLibrary Assay Design Center (http://www.roche-applied-science.com) and PCR products were detected using corresponding probes from the Universal ProbeLibrary (Roche) (Table 2). The glyceraldehyde-3-phosphate dehydrogenase (GAPDH) housekeeping gene was used as an endogenous internal control. All target genes were normalized using the housekeeping gene GAPDH, and analyzed with the 2^–△△Ct^ method. Changes in gene expression were expressed as fold-change.

**Table 2:** Primers used in the real-time polymerase chain reaction analysis

Target gene	Forward primer	Reverse primer	GenBank	Roche
			accession no.	probe no.
Housekeeping gene				
*GAPDH*	agccacatcgctcagacac	gcccaatacgaccaaatcc	NM_002046	#60
Anabolic genes				
*COLIA1*	gggattccctggacctaaag	ggaacacctcgctctcc	NM_000088	#67
*COLIIIA1*	ctggaccccagggtcttc	catctgatccagggtttcca	NM_000090	#20
*Aggrecan*	cctccccttcacgtgtaaaa	gctccgcttctgtagtctgc	NM_01135	#76
Catabolic genes				
*MMP-1*	gctaacctttgatgctataactacga	tttgtgcgcatgtagaatctg	NM_001145938.1	#7
*MMP-3*	caaaacatatttctttgtagaggacaa	ttcagctatttgcttgggaaa	NM_002422	#36
*ADAMTS-4*	ccaggcactgggctactact	gaacagggggtcccatcta	NM_005099	#67

*ADAMTS-4* A disintegrin and metallopeptidase with thrombospondin motif 4, *COLIA1* Collagen type IA1, *COLIIIA1* Collagen type IIIA1, *GAPDH* Glyceraldehyde-3-phosphate dehydrogenase, *MMP* Matrix metalloproteinase

The relative expression of different genes in the different groups was analyzed by one-way analysis of variance (ANOVA) with the Tukey’s post-hoc test. *P* < 0.05 indicated statistical significance.

## Results

**Fig. 4:**
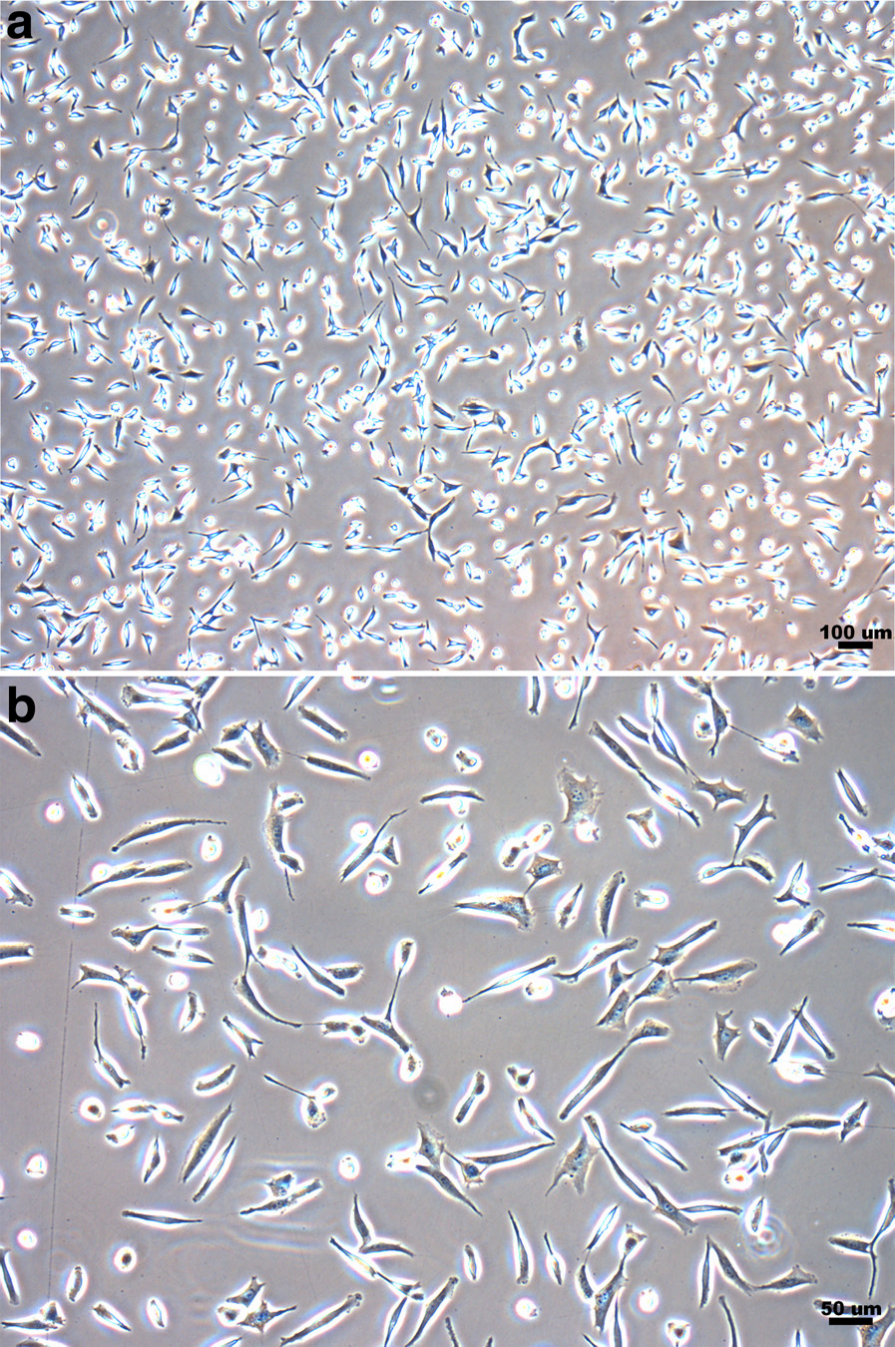
Morphology of hAF cells harvested from degenerated disc tissues. **a** The scale bar indicates 100 μm. **b** The scale bar indicates 50 μm. Cells were cultured for 20 days. Cells were spindle-shaped and plastic adherent. The doubling time was 63 ± 12 h (range, 52–78 h)

The disc tissues were classified as degeneration grades III or IV based on Pfirrmann’s classification [18]. When grown in monolayers, hAF cells harvested from degenerated disc tissues were astrocytes-like cells with one or three protrusions with a doubling time of 63 ± 12 h (range 52–78 h) (Fig. 4). A pre-test regarding biocompatibility in our custom-fabricated bio-microfluidic device was considered good enough to perform further experiments.

hAF cells were attached on the Fn-coated bio-microfludic device after overnight incubation at 37 °C, and had a similar morphology to cells cultured in a dish. When the cells were ready, the whole bio-microfluidic device was set up by assembling the laser-cut dishes with the microfluidic chips using vacuum force. Different levels of shear stress were applied (Figs. 3 and 4). Additionally, the morphology of cells seeded on the bio-microfluidic device remained unchanged after being subjected to 1 or 10 dyne/cm^2^ shear stress for 4 h.

**Fig. 5:**
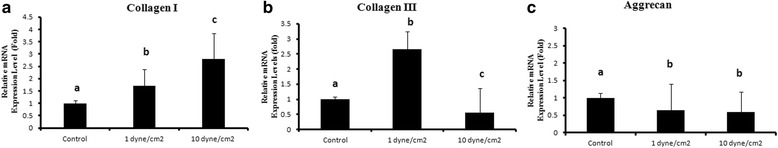
Semi-quantitative PCR for relative quantitation of ECM genes expression after 1 and 10 dyne/cm^2^ fluid-induced shear stress for 4 h. The control group (0 dyne/cm^2^) was not subjected to fluid-induced shear stress on the bio-microfluidic device. Semi-quantitative PCR was used to determine relative quantitative gene expression of **a** *COLI*, **b** *COLIII* and **c** *aggrecan*. Data represent mean ± standard deviation (n = 6). Differences were significant between groups with different letters (*P* < 0.05). Differences were not significant between groups with the same letter

**Fig. 6:**
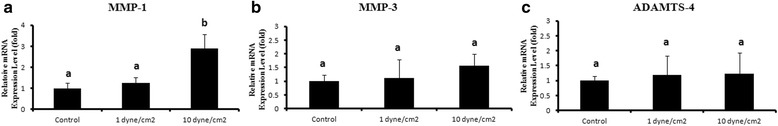
Semi-quantitative PCR for relative quantitation of metalloproteinase genes expression after 1 and 10 dyne/cm^2^ fluid-induced shear stress for 4 h. Semi-quantitative PCR was used to determine relative quantitative gene expression of **a** *MMP-1*, **b** *MMP-3* and **c** *ADAMTS-4*. Data represent mean ± standard deviation (n = 6). Differences were significant between groups with different letters (*P* < 0.05). Differences were not significant between groups with the same letter

We compared the expression of ECM and metalloproteinase genes in the cells subjected to shear stress with control cells (Figs. 5 and 6). Cells subjected to 1 and 10 dyne/cm^2^ shear stress had a significantly higher expression of *COLI* compared to control cells (1.72 ± 0.65- and 2.49 ± 1.06-fold higher, respectively), suggesting that the *COLI* gene was regulated in a stress-dependent manner. Expression of the *COLIII* gene was 2.65 ± 0.6 higher than control cells following 1 dyne/cm^2^ shear stress, but 0.57 ± 0.79-fold lower than the control group following 10 dyne/cm^2^ shear stress. Expression of the *COLIII* gene was significantly lower following 10 dyne/cm^2^ shear stress compared to cells subjected to 1 dyne/cm^2^. Cells subjected to 1 and 10 dyne/cm^2^ shear stress had a significantly lower expression of the *aggrecan* gene compared to the control group (0.65 ± 0.74- and 0.59 ± 0.58-fold lower, respectively). Expression of the *aggrecan* gene was also significantly lower following 10 dyne/cm^2^ shear stress compared to cells subjected to 1 dyne/cm^2^.

Expression of the *MMP-1* gene was 2.9 ± 0.76-fold higher in cells subjected to 10 dyne/cm^2^ shear stress compared to the control group. Expression of the *MMP-1* gene was similar between the control and 1 dyne/cm^2^ groups. There was no significant difference in the expression of the *MMP-3* and *ADAMTS-4* genes between the three groups.

The above results suggested that 1 dyne/cm^2^ exerted an anabolic effect on the *COLI* and *COLIII* genes but a catabolic effect on the *aggrecan* gene. It was also possible that 10 dyne/cm^2^ shear stress exerted an anabolic effect on the *COLI* gene but a catabolic effect on the *COLIII* and *aggrecan* genes.

## Discussion

hAF cells are exposed to shear stress during normal physiologic movement of the spine [3], and the mechanical stimulation is important for regulating homeostasis of the AF matrix [2, 3, 6–8, 10, 14]. The disc along with AF tissues which serves as a load-bearing structure is exposed to different types of daily recurring mechanic loads including tension, compression, and shear stress [22]. All these types of mechanical loading may occur simultaneously, resulting in a complex combination loading situation in vivo [22]. Disc cell types and species, loading types, frequency, magnitude and duration are among the key factors thought to play important roles in AF-ECM homeostasis [22].

*MMP-1*, which belongs to the subclass of collagenases, has been reported to be the most common MMP in the degenerated disc, with 91 % positive staining [23]. *MMP-1* activity is highest against collagen type III (> I > II) [23]. Collagen types I and III are distributed in the AF [9]. *MMP-3*, which plays a major role in disc degeneration, has a broad substrate specificity, and has been shown to degrade proteoglycans, lamin, Fn, gelatins, and collagen types II, III, IV, and V [23]. Degenerative disc is characterized by an upregulation of degradative enzymes such as MMPs, along with a declining ability to produce ECM [4, 5]. Both *MMP-1* and -3 have also been found in the AF in the degenerated disc, and this may contribute to the pathogenesis of AF matrix degeneration.

Besides type I and III collagens, the other major ECM component of the AF is proteoglycans, particularly *aggrecan*. *Aggrecan* is substituted with several negatively charged glycosaminoglycans (GAGs) which are responsible for its water-binding capacity [1, 5]. *ADAMTS-4* (aggrecanase-1), which is a key proteinase for the breakdown of *aggrecan*, has been shown to play an important role in the pathogenesis of AF degeneration. Several studies reported that mechanical stimuli may impact proteoglycan metabolism in hAF cells. *Aggrecan* gene expression was inhibited in hAF cells subjected to 10 % cyclic tensile strain at a frequency of 1 Hz for 24 h [17], while 0.4 MPa of compressive stress at 1 Hz for 2 h twice a day up to 7 days had catabolic effects on *aggrecan* gene expression [24]. Our results indicated that shear stress of 1 and 10 dyne/cm^2^ resulted in downregulation of *aggrecan* gene expression.

Shear stress of 1 and 10 dyne/cm^2^ also affected the expression of *COLI* in a stress-dependent manner, while the expression of the *MMP-3* and *ADAMT-4* genes remained unchanged in cells subjected to shear stress of 1 and 10 dyne/cm^2^ compared to the control group. It is possible that, in addition to *MMP-1*, -3 and ADAMT-4, other degradative enzymes or mechano-transduction pathways may be involved in the pathogenesis of ECM metabolism following shear stress. Shear stress of 1 dyne/cm^2^ seemed to have anabolic effects on the expression of the collagen I and III genes in degenerated hAF cells. However, a higher shear stress (10 dyne/cm^2^) exerted an anabolic effect on *COLI* expression, and a catabolic effect on *COLIII* and *aggrecan* expression.

Mechanically, AF cells bear shear stress during bending or twisting of the trunk; but few studies have investigated the effects of shear stress on the expression of different genes in hAF cells subjected to stress. hAF cells have been shown to respond to laminar shear stress by increasing intracellular calcium levels [3]. Our data differed from a previous report which showed that the expression of the *MMP-1* and -3 genes was upregulated in rabbit tendon cells following 1 dyne/cm^2^ fluid-induced shear stress for 6 h in a specially designed multi-slide flow device [25]. This difference could be due to species variation and differences in the magnitude of force at the cellular level.

Previous reports [11, 26] indicated that excessive loading such as heavy lifting, bending and twisting may play an important role in increased incidence of disc degeneration and herniation. Neidlinger-Wilke et al. [22] reported a baseline intradiscal pressure measuring around 0.1–0.2 MPa under conditions of supine rest, due to the combined effect of muscle loads and osmotic potential of the disc. Shear stress was estimated at 1600 NT at the L5–S1 disc level while stooping or weight lifting [27]. The shear stress applied in our study (1 and 10 dyne) was lower than that seen under physiological conditions. The reason for this was that AF cells may de-attach from the bio-microfludic in vitro following much higher shear stress. The in vitro situation is therefore different from that in vivo with muscles, ligaments and facet articular joint supports.

Our study had a number of limitations which should be considered. Firstly, our results were based on a cell-in-channel model, which did not reflect the real three-dimensional disc environment in vivo during body motion. Secondly, the magnitude of shear stress that hAF cells are subjected to during body motion still needs to be measured, which would validate our results. Thirdly, cells may be detached following fluid-induced shear stress in our chip, which may interfere with our results. Moreover, we still lacked the results of gene expression in healthy hAF cells harvested from discs in post-mortem young donors without prior systemic diseases or spine trauma, which could provide the real control results and allow us to compare between healthy and degenerated discs to understand the role of shear stress in the pathogenesis of disc degeneration.

In summary, we demonstrated that the expressions of ECM and metalloproteinase genes in hAF cells were influenced by fluid-induced shear stress. Although not all aspects of alterations of the mechanical environment of the disc can be extrapolated from the in vivo situation, our results may provide better understanding of the physiology of the degenerated discs and the pathogenesis of disc degeneration following shear stress.

## Conclusion

Fluid-induced shear stress regulated ECM and MMP genes expression in hAF cells. The study may clarify the role of shear stress in the patho-mechanism of disc degeneration as well as the development of a new therapeutic strategy for IVD degeneration.
